# Mannose-Coated Reconstituted Lipoprotein Nanoparticles for the Targeting of Tumor-Associated Macrophages: Optimization, Characterization, and In Vitro Evaluation of Effectiveness

**DOI:** 10.3390/pharmaceutics15061685

**Published:** 2023-06-08

**Authors:** Akpedje S. Dossou, Morgan E. Mantsch, Ammar Kapic, William L. Burnett, Nirupama Sabnis, Jeffery L. Coffer, Rance E. Berg, Rafal Fudala, Andras G. Lacko

**Affiliations:** 1 Department of Microbiology, Immunology and Genetics, UNT Health Science Center (UNTHSC), Fort Worth, TX 76107, USA; akpedjedossou@my.unthsc.edu (A.S.D.); ammarkapic@my.unthsc.edu (A.K.); nirupama.sabnis@unthsc.edu (N.S.); rance.berg@unthsc.edu (R.E.B.); 2 College of Natural Sciences, University of Texas at Austin, Austin, TX 78705, USA; morgan.mantsch@austin.utexas.edu; 3 College of Science and Engineering, Texas Christian University (TCU), Fort Worth, TX 76129, USA; w.l.burnett@tcu.edu (W.L.B.); j.coffer@tcu.edu (J.L.C.)

**Keywords:** DMXAA, rHDL NPs, mannose, TAMs, ID8, RAW 264.7, Nile Red, M1, M2

## Abstract

Reconstituted high-density lipoprotein nanoparticles (rHDL NPs) have been utilized as delivery vehicles to a variety of targets, including cancer cells. However, the modification of rHDL NPs for the targeting of the pro-tumoral tumor-associated macrophages (TAMs) remains largely unexplored. The presence of mannose on nanoparticles can facilitate the targeting of TAMs which highly express the mannose receptor at their surface. Here, we optimized and characterized mannose-coated rHDL NPs loaded with 5,6-dimethylxanthenone-4-acetic acid (DMXAA), an immunomodulatory drug. Lipids, recombinant apolipoprotein A-I, DMXAA, and different amounts of DSPE-PEG-mannose (DPM) were combined to assemble rHDL-DPM-DMXAA NPs. The introduction of DPM in the nanoparticle assembly altered the particle size, zeta potential, elution pattern, and DMXAA entrapment efficiency of the rHDL NPs. Collectively, the changes in physicochemical characteristics of rHDL NPs upon the addition of the mannose moiety DPM indicated that the rHDL-DPM-DMXAA NPs were successfully assembled. The rHDL-DPM-DMXAA NPs induced an immunostimulatory phenotype in macrophages pre-exposed to cancer cell-conditioned media. Furthermore, rHDL-DPM NPs delivered their payload more readily to macrophages than cancer cells. Considering the effects of the rHDL-DPM-DMXAA NPs on macrophages, the rHDL-DPM NPs have the potential to serve as a drug delivery platform for the selective targeting of TAMs.

## 1. Introduction

Macrophages located in the tumor microenvironment (TME) are termed tumor-associated macrophages (TAMs). TAMs represent one of the most abundant immune cell populations in the TME and are derived from tissue-resident macrophages and tumor-infiltrating monocytes [1]. While TAMs are characterized by phenotypic heterogeneity, the immunosuppressive (M2-like) and the immunostimulatory (M1-like) are subpopulations influencing the most significant impact on tumor progression [2,3,4]. Components of the TME, including cancer cells and secreted factors, induce an M2-like phenotype in TAMs [5,6,7,8]. In several cancer types, M2-like TAMs predominate in the TME and promote immunosuppression, tumor growth, metastasis, and resistance to cancer treatments, including chemotherapy and immunotherapy [4,9,10]. In contrast, M1-like TAMs have been reported to facilitate the intratumoral infiltration of cytotoxic immune cells, enhance the anticancer effects of clinical therapies, and are associated with favorable survival prognosis [11,12,13]. Therefore, several strategies have been devised to reprogram TAMs from the M2-like to the M1-like phenotype. Among these reprogramming strategies, agonists of the Stimulator of Interferon genes (STING) have shown promising antitumoral effects across several cancer types [14,15,16]. However, when the M2-to-M1 reprogramming agents are freely available in systemic circulation, the ubiquitous distribution of macrophages in the body increases the possibility of immune adverse events [17]. Moreover, the complex architecture of the TME and the availability of M2-to-M1 reprogramming agents to the multiple cell types in the TME can decrease their accessibility to TAMs and thus reduce their effectiveness [18]. Nanoparticles equipped with the fine-tuned release of their content can help address these challenges through the specific targeting of TAMs in the delivery of M2-to-M1 reprogramming agents in the TME [19]. While TAMs have the ability to phagocytose nanoparticles introduced in the TME [20], functionalized nanoparticles that target highly expressed surface receptors, such as the mannose receptor, have been particularly effective in the specific targeting of TAMs [21].

High-density lipoproteins (HDL) constitute a heterogenous class of lipoproteins involved in mammalian cholesterol homeostasis [22,23]. Composed of lipids and proteins, HDL particles occur endogenously with a mean diameter size range of 7–12 nm [24]. They can transport and deliver cholesterol as well as a variety of hydrophobic biomolecules to target tissues, with their structural proteins—also called apolipoproteins—serving to recognize target cells [25]. Owing to the natural affinity of apolipoproteins to phospholipids [26], the main individual components of HDL can be reassembled in vitro to form the reconstituted HDL nanoparticles (rHDL NPs) [27,28]. With regard to the anti-atherogenic, immunomodulatory, and transport capabilities of HDL particles [29,30], the rHDL NPs have been utilized either unloaded or loaded with drugs, imaging agents, or biomolecules to target a variety of cells, including macrophages, mostly in the context of addressing cardiovascular pathologies [31,32,33,34] and cancer cells [35,36,37]. While studies have shown that rHDL NPs are taken up by TAMs among other cells in the TME [38,39], much has yet to be explored regarding the use of functionalized rHDL NPs for the specific targeting of TAMs. 

In this study, we optimized the physicochemical characteristics of mannose-coated rHDL NPs loaded with 5,6-dimethylxanthenone-4-acetic acid (DMXAA), a murine STING agonist [40]. Since 1,2-distearoyl-sn-glycero-3-phosphoethanolamine-N-(polyethyleneglycol)-Mannose (DSPE-PEG-Mannose, referred to as DPM) is an amphipathic molecule with a large hydrophilic moiety ending with mannose, we hypothesized that it would be incorporated into the rHDL NPs, forming the mannose-coated rHDL NPs (rHDL-DPM NPs). The rHDL-DPM-DMXAA NPs displayed adequate nanoparticle characteristics, and changes in particle size, zeta potential, elution patterns, and DMXAA steady-state anisotropy indicate that both the DPM and DMXAA are associated with the rHDL NPs. We also evaluated payload delivery via the rHDL-DPM NPs and the biological activity of rHDL-DPM-DMXAA NPs in murine RAW 264.7 macrophages pre-exposed to murine ovarian cancer cell (ID8) conditioned media. The rHDL-DPM-DMXAA NPs effected an M2-to-M1 phenotype reversal and elicited secretion of the antitumoral cytokine interferon beta (IFNβ) in ID8 conditioned medium (CM)-educated macrophages. Furthermore, the rHDL-DPM NPs could modulate macrophage functional phenotype and deliver payload more efficiently to the ID8 CM-educated macrophages than to ID8 cells. Together, the findings from this study highlight the feasibility of the assembly of the mannose-coated rHDL NPs and the potential therapeutic benefit that might be gained from their application in the specific targeting of TAMs. 

## 2. Materials and Methods

### 2.1. Materials

The egg yolk L-α-phosphatidyl choline (EYPC, product # 61755), free cholesterol (FC, product # C8667), block lipid transport-1 (BLT-1, product # 373210), Nile Red (NR, product # 72485), ID8 mouse ovarian epithelial cancer cells (product # SCC145), and other organic and inorganic chemicals (unless otherwise stated) were purchased from Sigma-Aldrich Corporation (St Louis, MO, USA). The apolipoprotein A-I (ApoA-I) was produced endotoxin-free in Chinese hamster ovarian (CHO) cells and was provided by Cerenis Therapeutics, now Abionyx Pharma (Balma, France). The 1,2-distearoyl-sn-glycero-3-phosphoethanolamine-polyethylene glycol (2000)-mannose (DSPE-PEG(2K)-mannose or DPM, Cat# LP096282, Cat ID: 12169) was purchased from Biopharma PEG Scientific Inc (Watertown, MA, USA). The DSPE-PEG (Cat# MPL0301) was purchased from Advanced BioChemicals, LLC (Lawrenceville, GA, USA). The mouse Interferon gamma-induced protein 10 (IP-10, also called the C-X-C Motif Chemokine Ligand 10, CXCL10) ELISA kit (Cat# BMS6018), Hoechst nuclear stain solution (Cat# 62249), general research and cell culture supplies (unless otherwise stated) were obtained from Thermo Fisher Life Technologies Corporation (Carlsbad, CA, USA). The mouse tumor necrosis factor-alpha (TNFα) ELISA kit (Cat# MTA00B), mouse interleukin 10 (IL-10) ELISA kit (Cat# M1000B), mouse IFNβ ELISA kit (Cat# MIFNB0), vascular endothelial growth factor (VEGF) ELISA kit (Cat# MMV00) were supplied by R&D Systems (Minneapolis, MN, USA). The 5,6-dimethylxanthenone-4-acetic acid (DMXAA, also called Vadimezan, Cat# HY-10964) was purchased from MedChemExpress (Monmouth Junction, NJ, USA). RAW 264.7 mouse macrophages (TIB-71) and the H9C2 cardiomyocytes (CRL-1446) were purchased from the American Type Culture Collection (ATCC, Manassas, VA, USA). The primary human astrocytes were donated by Dr. Kathleen Borgmann. The uranyl acetate (depleted uranium, Cat# 02624-AB) and the formvar carbon-coated 200 mesh copper grids (Cat# 3420C-CF) were supplied by Structure Probe Inc. (West Chester, PA, USA).

### 2.2. Synthesis and Optimization of Mannose-Coated rHDL NPs

The assembly of the nanoparticles was performed as previously described [41,42,43] with some modifications. Briefly, free cholesterol (FC), egg yolk phosphatidylcholine (EYPC), and DSPE-PEG-mannose (DPM) were individually dissolved in chloroform (Sigma-Aldrich). Then, FC, EYPC, and DPM solutions were mixed in scintillation glass vials and dried to a thin film under a stream of nitrogen gas to remove the chloroform. The thin film was then rehydrated with phosphate-buffered saline (PBS), pH 7.4 (Thermo Fisher Life Technologies Corporation, Cat# 10010), and vortexed until the formation of a uniform milky suspension of lipids. Depending on the type of 1× formulation (empty or loaded nanoparticles), the payload (Nile red dye or DMXAA) was added in powder form to the thin film before rehydration. The lipid/payload suspension was sonicated with probe immersion with an ultrasonic processor (QSonica, Item # UX-04712-51, Cole-Palmer, Vernon Hills, IL, USA) for 30 min on ice (2 min, six times, with 3 min of rest intercalating between sonication bursts) at amplitude 80. After sonication, ApoA-I in 6 M guanidine hydrochloride was added dropwise to the lipid emulsion with gentle stirring. After overnight incubation with shaking at 4 degrees Celsius in the dark, the mixture of ApoA-I, EYPC, FC, and DPM with or without payload was dialyzed against PBS for 6 h using a 50 KDa dialysis bag to remove unbound ingredients. After dialysis, the preparations were centrifuged at 12,000 rpm at 4 degrees Celsius for 30 min to remove large aggregates, debris, and unbound payload. Finally, the preparations were filtered through a sterile 0.2 µm syringe filter and stored at 4 degrees Celsius in the dark until physicochemical characterization and in vitro effectiveness studies. A molar ratio of 1:100:12 (ApoA-I:EYPC:FC) was utilized for all rHDL NPs preparations. For the optimization of the physicochemical characteristics of the mannose-coated rHDL nanoparticles, different ratios of DPM to EYPC were utilized while keeping the amount of all the other ingredients constant. For comparison purposes, particles assembled without DPM were included in the study and are referred to as No DPM. The empty formulations with DPM are referred to as rHDL-DPM NPs, while the formulations with DMXAA and the Nile Red (NR) dye are referred to respectively as rHDL-DPM-DMXAA NPs and rHDL-DPM-NR NPs. Freshly prepared and characterized nanoparticles were utilized for all studies conducted.

### 2.3. Characterization of Nanoparticles

#### 2.3.1. Physical Characterization

The particle diameter size, polydispersity index, particle concentration, and zeta potential of all the formulations were acquired using the light scattering system Zetasizer Ultra (Malvern Panalytical Ltd., Malvern, UK). For dynamic light scattering (DLS), the particles were 20-fold diluted in an aqueous buffer (PBS, 0.2 µM pre-filtered) and transferred to a spectrophotometer cuvette. To assess the zeta potential, folded capillary cells (product # DTS1070, Malvern Panalytical Ltd., Malvern, UK) were used instead. The data was analyzed using the ZS Xplorer software (Malvern Panalytical Ltd., Malvern, UK). Each experimental replicate measurement is acquired as an average of three measurement runs of the sample on the instrument. Transmission electron microscopy (TEM) using negative staining was also used to confirm the size and shape of the particles. Briefly, the samples were 1000-fold diluted in deionized water. Then, 10 µL of the diluted sample was pipetted onto the carbon side of the discharged grid and left to settle for 2 min, after which the excess sample was blotted out with filter paper, and 10 µL of nanopure water was added to the grid. The excess water was blotted out after 2 min and replaced by 2% uranyl acetate. After 2 min of incubation, the uranyl acetate was blotted out, and the sample was left to dry overnight in the grid box. The images were acquired at 200 keV using the JEM-2100 (Jeol Ltd., Peabody, MA, USA). Both the sample and the uranyl acetate solution were filtered through a 0.2 µM pore size filter before addition to the formvar carbon-coated 200 mesh copper grids.

#### 2.3.2. Chemical Composition

The phospholipid content of the particles was acquired using colorimetric enzymatic assays of the Wako phospholipid C assay (Wako Diagnostics Life Sciences, Richmond, VA, USA). The ApoA-I content was assessed via bicinchoninic acid assay (Thermo Fisher Life Technologies Corporation, Cat# PI23225). The DPM content was determined using a barium chloride/iodide assay, which relies on the color change that occurs upon the chelation of PEG as BaI_2_ crystals form from the reaction of BaCl_2_ and I_2_. The BaCl_2_/I_2_ assay was performed as outlined previously [44,45] with some modifications. Briefly, 50 µL of each nanoparticle solution to be characterized was deproteinized via a 1:1 dilution in 1 M HCl. After 10 min of incubation at room temperature, the mixture was centrifuged at 5000 rpm for 5 min to remove the denatured protein. Then, 25 µL of supernatant was pipetted onto a 96-well plate with the sequential addition of 100 µL of 2.5% BaCl_2_ and 25 µL of 0.005 N iodine solution. Standards of increasing DPM concentration ranging from 0 to 500 µg/mL were also included in the assay. The absorbance measurement of the color change was immediately read at 450 nm using the spectrophotometer plate reader function of the Biotek Cytation 3 imaging reader (Agilent Technologies Inc., Santa Clara, CA, USA). The DMXAA concentration was estimated via absorbance (350 nm) measurements via a spectrophotometer with baseline correction with PBS. The absorbance of DMXAA was calculated by subtracting the absorbance profile of the DPM-loaded nanoparticles from the scattering absorbance profile of the empty nanoparticles. Concentrations of free DMXAA (DMXAA dissolved in 7.5% sodium bicarbonate) ranging from 0 to 500 µg/mL were used as standards.

#### 2.3.3. Entrapment Efficiency

After the determination of the DMXAA and DPM content, respectively, via absorbance and the BaCl_2_/I_2_ assay, the equation (Equation (1)) below was used to determine the entrapment efficiency (EE) of both the DMXAA and the DPM.

(1) $$\;\mathrm{EE}\;(\%)=\frac{\mathrm{Mass}\;\mathrm{of}\;\mathrm{DMXAA}\;\mathrm{in}\;\mathrm{purified}\;\mathrm{nanoparticles}}{\mathrm{Mass}\;\mathrm{of}\;\mathrm{DMXAA}\;\mathrm{initially}\;\mathrm{added}\;\mathrm{to}\;\mathrm{the}\;\mathrm{formulation}}\;\times \;100$$

#### 2.3.4. Fast-Protein Liquid Chromatography

To assess the effect of DPM addition to the rHDL NPs on particle elution pattern and further confirm the stable assembly of the rHDL-DPM-DMXAA NPs, the particles, as well as human HDL, were analyzed with fast-protein liquid chromatography (FPLC) using the Amersham Pharmacia AKTA FPLC chromatography system (GE Healthcare Technologies, Chicago, IL, USA) with protein detection at absorbance 280 nm. The samples were run through a Cytiva Superose 6 Increase 10/300 column (Sigma Aldrich, product # GE29-0915-96) at a flow rate of 0.5 mL/min with PBS as the elution buffer. The results were analyzed using the UNICORN 5 software (Cytiva, Marlborough, MA, USA).

#### 2.3.5. Steady-State Anisotropy

To assess changes in steady-state anisotropy (*r*) from the free payload (DMXAA and NR) to their rHDL-DPM formulation counterparts, the samples were transferred into a quartz cuvette with a 10 mm light path and a 2 mm light width (Science Outlet, Tsuenwan, Hong Kong). Fluorescence measurements were carried out using a manual light polarizer (Varian 00-100761-00) fitted to a fluorescence spectrophotometer (Varian Cary Eclipse, Varian Inc., Palo Alto, CA, USA). The steady-state anisotropy (*r*) for all fluorophores in this study (DMXAA and Nile Red) was calculated using the following equation (Equation (2)):

(2) $$r=\frac{I_{\mathrm{vv}}-{\;\mathrm{GI}}_{\mathrm{vh}}}{I_{\mathrm{vv}}-{\;2\mathrm{GI}}_{\mathrm{vh}}}\;$$

where “*I*” is the fluorescence intensity acquired at different combinations of the positioning of the polarizer at the excitation and the polarizer at the emission. The first subscript denotes the vertical (*v*) or horizontal (*h*) placement of the polarizer at the excitation while the second subscript refers to the placement of the polarizer at the emission. The *G* factor is the ratio of *I_hv_* to *I_hh_*. Excitation 350 nm, emission 415 nm was used for DMXAA (short-pass filter 400 nm, long-pass filter 400 nm) was used for DMXAA. The free NR is Nile Red dissolved in DMSO (excitation 550 nm, emission 635 nm, short-pass filter 595 nm, long-pass filter 600 nm). For particle disruption studies, the particles were dissolved 10-fold in DMSO and centrifuged (5000 rpm, 3 min, 4 degrees Celsius) to remove protein aggregates, and the supernatant was analyzed.

### 2.4. In Vitro Drug Release Assessment

To assess the ability of the nanoparticles to retain the payload and prevent early release, 2 mL of the 1:50 rHDL-DPM-NR samples were placed into a 50 kDa dialysis bag immersed in 50 mL PBS buffered supplemented with 2% (*v*/*v*) Tween-20 to facilitate the exit of the free dye from the dialysis bag. An equivalent amount of free Nile Red dissolved in 1:1 DMSO: water was placed in a separate dialysis bag for comparison. The bags were left to incubate at 37 degrees Celsius with shaking at 150 rpm in the dark. Every hour, 1 mL of the surrounding buffer was collected and replaced with fresh 1 mL buffer. The fluorescence (excitation 550 nm, emission 635 nm) of the collected buffer (200 µL of the sample in a black 96-well plate) was measured using the fluorescence mode of the Biotek Cytation 3 imaging reader. As a reference, 2 mL of the rHDL-DPM-NR and the free NR were directly added in 50 mL buffer, and the fluorescence intensity (excitation 550 nm, emission 635 nm) recorded served as the 100% dye released measurement. The following equation (Equation (3)) was used to calculate the percent dye released:

(3) $$\mathrm{Percent}\;\mathrm{dye}\;\mathrm{released}=\frac{\mathrm{NR}\;\mathrm{fluorescence}\;\mathrm{recorded}\;\mathrm{at}\;\mathrm{the}\;\mathrm{time}\;\mathrm{point}}{\mathrm{Theoretical}\;\mathrm{fluorescence}\;\mathrm{of}\;100\%\;\mathrm{dye}\;\mathrm{released}}\;\times \;100$$

### 2.5. In Vitro Studies

#### 2.5.1. Cell Culture Conditions and Treatments

The RAW 264.7 macrophages, ID8 ovarian cancer cells, H9C2 cardiomyocytes, and astrocytes were cultured throughout the treatments at 37 degrees Celsius in 5% CO2 in a humidified incubator. Both the RAW 264.7 cells and H9C2 cardiomyocytes were maintained in complete DMEM (DMEM media supplemented with 10% FBS and 1% Pen Strep), while the ID8 cells were maintained in DMEM supplemented with 4% FBS and 1% Pen Strep. The astrocytes were maintained in cDMEM/F-12 media. The cell lines were routinely tested for mycoplasma contamination using the MycoFluor™ Mycoplasma Detection Kit and found to be negative throughout the study. To simulate TAMs, RAW 264.7 cells were exposed to ID8 conditioned medium (CM). Briefly, 2 × 10^6^ ID8 cells were seeded in T-75 flasks and maintained in complete DMEM (cDMEM) until 80–90% confluency. Then, the culture media was collected and centrifuged twice at 1500 rpm for 5 min to remove cellular debris. The resulting supernatant was filtered through a sterile 0.45µM-pore size filter and stored at −80 degrees Celsius until use. To generate CM-educated macrophages, 2 × 10^6^ RAW 264.7 cells were seeded in a 60 mm dish and were allowed to attach overnight. Then, the seeded RAW 264.7 cells were maintained in cDMEM supplemented with 20% of ID8 CM for up to 48 h. The ID8 CM-educated RAW 264.7 cells were treated for 24 h with an equivalent amount of 20 ug/mL DMXAA for free DMXAA, rHDL-DPM-DMXAA NPs, and the associated controls, which include the vehicle (7.5% sodium bicarbonate), rHDL-DPM NPs. All formulations used for cellular treatments were sterilized via filtration through a sterile 0.2 µm-syringe filter. Due to the low DMXAA EE% observed with the rHDL-DMXAA NPs, these particles were not included in the study. 

#### 2.5.2. Cytotoxicity Studies

The direct effect of the nanoparticles on untreated RAW 264.7 cells, ID8-educated RAW 264.7 cells, ID8 cells, H9C2 cardiomyocytes, and astrocytes was assessed using the cytotoxicity CCK8 Kit (Dojindo Molecular Technologies, Tubaru, Japan) following the manufacturer manual. Briefly, 5 × 10^3^ cells were seeded in a 96-well plate and incubated in their respective media overnight. Then, the cells were either left untreated or treated with vehicle, free DMXAA, rHDL-DPM NPs, and rHDL-DPM-DMXAA NPs for a total final volume of 100 µL. After 24 h of incubation, 10 µL of the CCK8 kit reagent was added to the wells with incubation at 37 degrees Celsius, and the developed formazan dye color was monitored and read within 2 h at 450 nm using the Biotek Cytation 3 plate reader. The cytotoxicity results are presented as percent absorbance at 450 nm. After absorbance values are corrected with controls, the percent absorbance is calculated as follows (Equation (4)):

(4) $$\mathrm{Percent}\;\mathrm{absorbance}\;\mathrm{at}\;450\;\mathrm{nm}=\frac{\mathrm{Absorbance}\;\mathrm{at}\;450\;\mathrm{nm}\;\mathrm{recorded}\;\mathrm{for}\;\mathrm{treatment}}{\mathrm{Absorbance}\;\mathrm{at}\;450\;\mathrm{nm}\;\mathrm{of}\;\mathrm{untreated}\;\mathrm{control}}\;\times \;100$$

#### 2.5.3. Enzyme-Linked Immunosorbent Assays

After 24 h of incubation, the media from the different treatment groups of cells, as described in the above sections, were centrifuged (5000 rpm, 5 min, 4 degrees Celsius) to remove cellular debris. The supernatants were stored at −80 degrees Celsius until quantification of cytokines via enzyme-linked immunosorbent assay (ELISA). The supernatants of the same samples were assayed for TNFα, IFNβ, CXCL10 (IP-10), VEGF, and IL-10 following the kit manufacturer’s guidelines. The incubation media were included in the controls for all ELISA experiments. The values obtained from the ELISAs were normalized to cell numbers. 

#### 2.5.4. Uptake Studies

To investigate the effects of rHDL NPs on payload uptake, the ID8 CM-educated RAW 264.7 cells and ID8 cells were treated as previously described [46,47] with some modifications. Briefly, 2 × 10^5^ cells (RAW 264.7 cells, ID8 cells) were seeded in a poly-D-lysine-coated 35 mm glass-bottom dish and allowed to attach overnight. The RAW 264.7 cells were treated with ID8 CM, as described above. Before the uptake studies, the cells were rinsed twice with PBS and incubated either with the free NR or with the 1:50 rHDL-DPM-NR (with an equivalent amount of 0.5 µM NR) dispersed in cDMEM. Then, the cells were incubated for 5 min, 10, 15, 30 min, and 60 min. After incubation with the NR formulations, the cells were washed 3 times with PBS and either incubated with 5 µM Hoechst nuclear stain in PBS for 10 min, followed by 3 washes of PBS and re-incubated in phenol red-free DMEM media. The visualization of the cells and the NR mean fluorescence intensity (MFI) analysis per cell were performed using the Biotek Cytation Image reader and its cellular analysis features. The same exposure settings (intensity, integration time, camera gain) were utilized for all treated cells within an experiment to allow comparison between treatments.

### 2.6. Statistical Analysis 

The optimization of the rHDL-DPM-DMXAA NPs with different amounts of DPM was replicated in six independent studies. Unless otherwise stated, the data presented for other studies are representative of at least three independent experiments. All data were analyzed using the OriginPro 2022b software (OriginLab Corp., Northampton, MA, USA). Comparisons between the two groups were performed using the unpaired two-tailed Student’s *t*-test. A One-way ANOVA followed by a Tukey’s test was used to evaluate the statistically significant differences in treatment responses when more than two treatment groups were involved in the comparison. The statistical significance was evaluated at *p* < 0.05. Relevant non -significant differences are indicated as “n.s.” on the graphs. The results are presented as mean ± standard deviation (SD).

## 3. Results

### 3.1. The DPM Alters the Size and Surface Electrical Characteristics of the rHDL NPs

**Scheme 1:**
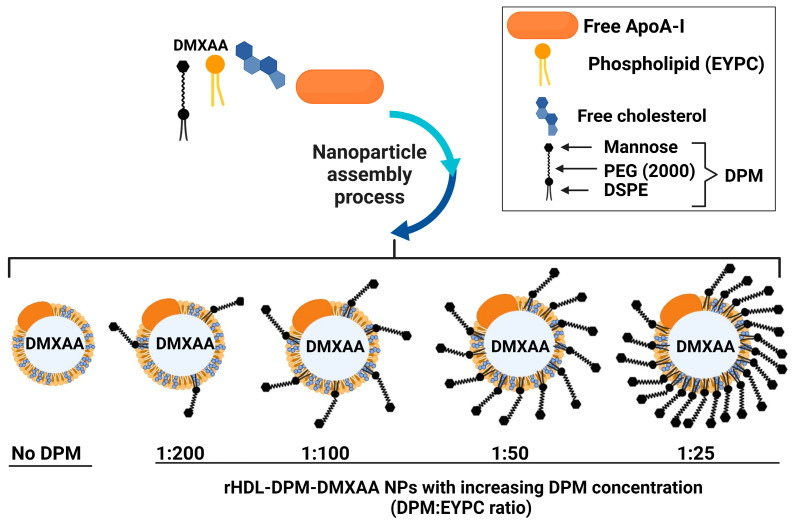
Optimization of the rHDL-DPM-DMXAA NPs. While the amounts of EYPC, free cholesterol, and ApoA-I were kept constant, varying amounts of the DPM were tested in the study to optimize response variables such as particle diameter size, polydispersity index, zeta potential, DPM, and DMXAA loading—image created with BioRender.com.

Different amounts of DPM (1:200, 1:100, 1:50, 1:25, DPM:EYPC ratio in the nanoparticles) were first tested to obtain particles that have maximal DPM and DMXAA loading capacity, as well as desirable physical characteristics (minimum particle diameter size and polydispersity index, negative zeta potential) for the cellular studies. The resulting formulations were characterized (Scheme 1). These DPM:EYPC were selected based on previous findings with mannosylated liposomes by other investigators [48] and on the number of lipid molecules (about 160 up to 300) per particle uncovered by both optimization studies and computational modeling studies of HDL-type particles [41,49,50]. 

**Figure 1:**
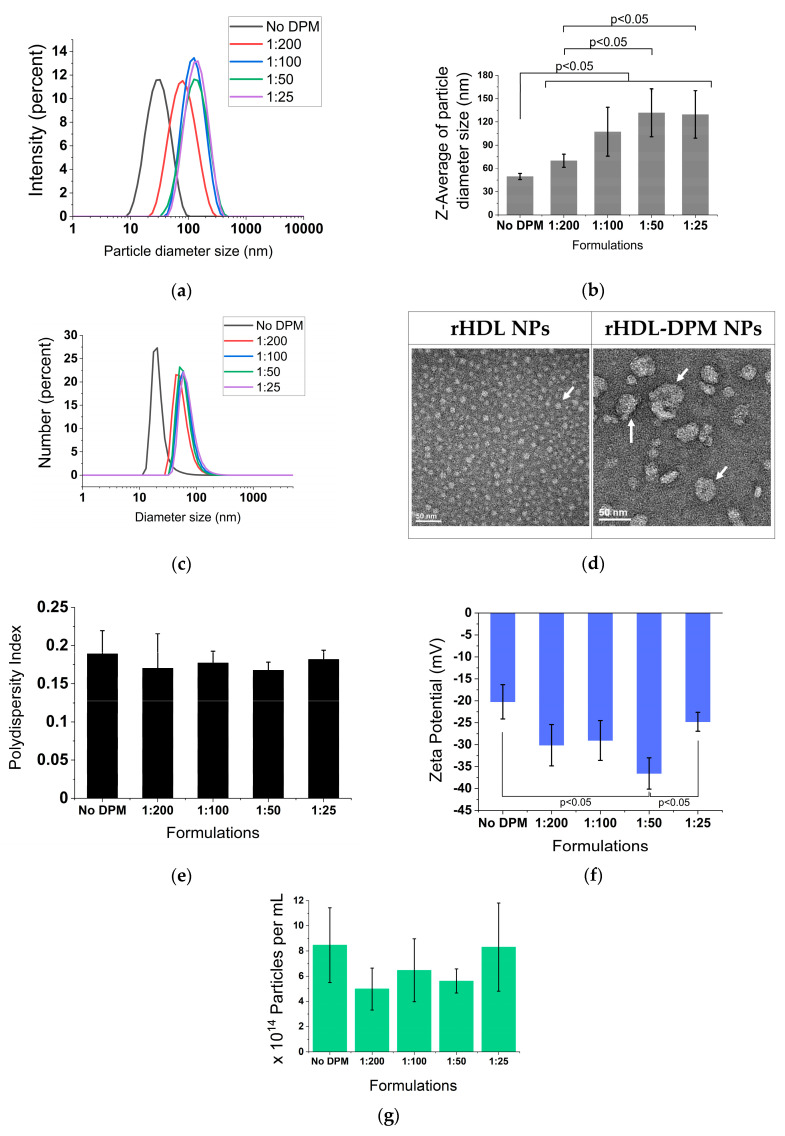
DPM alters the particle size and zeta potential of rHDL NPs. (**a**) Particle size distribution by the intensity of the formulation with or without DPM (No DPM). DPM (DSPE-PEG-mannose) ratios indicate the DPM to phospholipid ratio in the formulations; (**b**) z-average of particle size; (**c**) number distribution of particle size; (**d**) TEM images of rHDL NPs (340,000×) and the 1:50 rHDL-DPM NPs (350,000×)—scale bars: 50 nm. White arrows indicate particles; (**e**) polydispersity index of the formulations; (**f**) zeta potential of the formulations; (**g**) particle concentration. The data are representative of six independent experiments and are graphed as mean ± SD.

The DLS analysis via intensity distribution revealed a larger hydrodynamic diameter size for the rHDL-DPM-DMXAA NPs (up to 131.7 ± 31 nm) compared with their No DPM counterpart (49.5 ± 4 nm) (Figure 1a). This increase in hydrodynamic diameter size was dependent upon the DPM amount added in the formulation, with no significant differences between the 1:50 and 1:25 formulations (Figure 1b). Although the number distribution assessment yielded values nearly half the particle size estimated by intensity distribution, it also mirrored the increase in diameter size (Figure 1c). The particle images obtained from the TEM show spherical particles for both the rHDL-DPM NPs and their No DPM counterpart. Although the particle diameter sizes appear smaller with the TEM (less than 30 nm for the rHDL NPs and less than 100 nm for the rHDL-DPM NPs) compared to the intensity distribution values acquired via DLS, their presence of larger particles in the rHDL-DPM-DMXAA NPs group corroborates the increase in particle diameter size upon addition of the DPM (Figure 1d). The different nanoparticle preparations displayed a polydispersity index of less than 0.3 with no significant differences in the formulations (Figure 1e). All rHDL-DPM particles presented a negative zeta potential, with the 1:50 formulation exhibiting a significantly more negative zeta potential (−36.56 ± 3.6 mV) than other formulations, including the 1:25 formulation (Figure 1f). There were no statistically significant differences in particle concentration (at least 3 × 10^14^ particles per mL for all preparations, Supplemental Table S1), although the rHDL-DPM tended to have a slightly lower particle concentration than rHDL NPs (Figure 1g). Together, these data suggest that the addition of the DPM in the formulation of rHDL NPs produces an increase in particle size and can modify the zeta potential of the particles. 

### 3.2. The DPM Is Associated with the rHDL NPs

**Figure 2:**
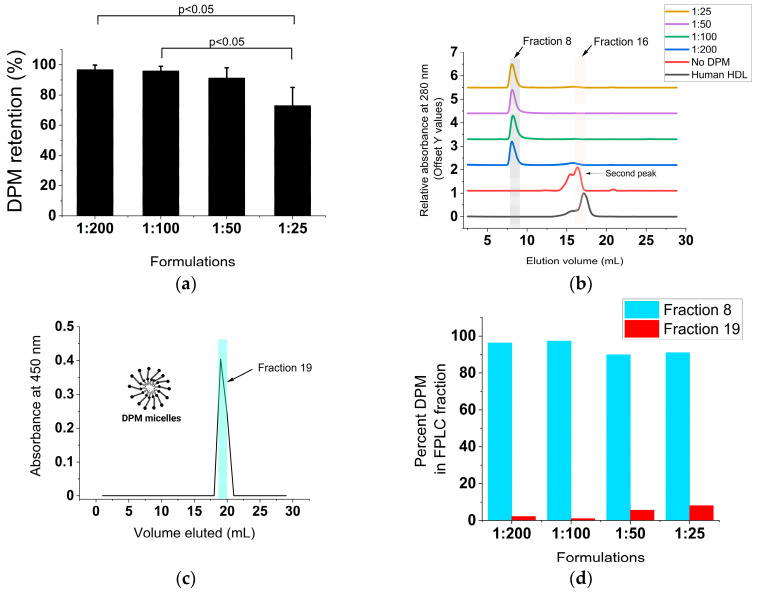

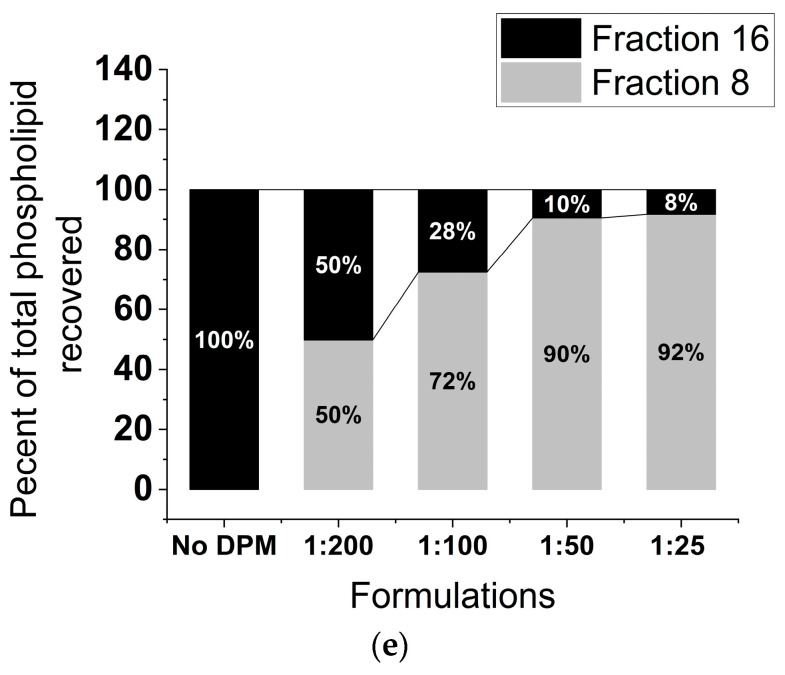
The DPM is associated with the rHDL NPs. (**a**) Percent DPM retained relative to initial DPM added during the assembly of the rHDL-DPM NPs, n = 6; (**b**) FPLC elution profile of the formulations, including normal human HDL. Fraction 8 and Fraction 16 are indicated for reference, representative data of six independent experiments; (**c**) FPLC elution profile of DPM micelles. Fraction 19 is indicated for reference, representative data of three independent experiments; (**d**) percent of total DPM recovered after elution of the different formulations through the FPLC column, representative of one experiment; (**e**) percent phospholipids recovered in the FPLC fractions of the different rHDL-DPM NPs, n = 6. The data are graphed as mean ± SD.

Nearly all the initial DPM added was retained in the different formulations, with the 1:25 rHDL-DPM-NPs showing a trend toward lower retention (73 ± 12% of initial DPM retained) (Figure 2a). The FPLC elution profile of the rHDL NPs, which usually resembles that of the normal human HDL, was altered with the addition of the DPM, with the elution peak appearing earlier (Figure 2b). DPM micelles were run on the FPLC column to confirm the association of the DPM with the rHDL NPs as opposed to the coexistence of DPM micelles and rHDL NPs in the same preparation. We hypothesized that due to the much lower molecular weight of the DPM micelles, they would elute later than rHDL NPs since the Superose 6 column is a size exclusion column. While the DPM micelles did not produce a signal at 280 nm, the BaCl_2_/I_2_ assay conducted on all eluted fractions revealed that DPM eluted at around 19 mL (also called Fraction 19), which is ~3 mL later than the standard 16 mL of the typical rHDL NPs on the same FPLC run settings (Figure 2c). In contrast to the late elution of DPM micelles, nearly all the DPM in the rHDL-DPM NPs eluted with the particles at Fraction 8, not at Fraction 19 (Figure 2d). Similar to the DPM, the protein component of the particles (absorbance 280 nm) and most of the phospholipids also eluted at Fraction 8 (Figure 2e). Collectively, these results suggest that the DPM is associated with the rHDL NPs. 

### 3.3. Addition of DPM to the rHDL NPs Improves DMXAA Retention in the Nanoparticles

**Figure 3:**
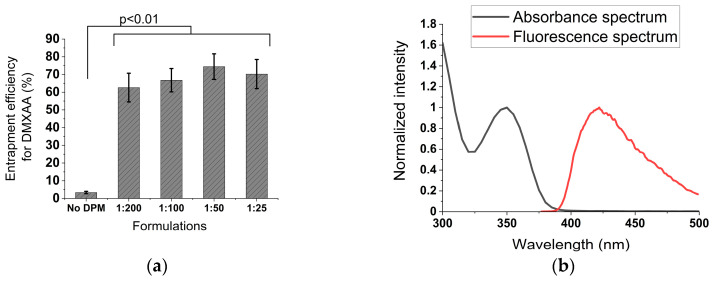

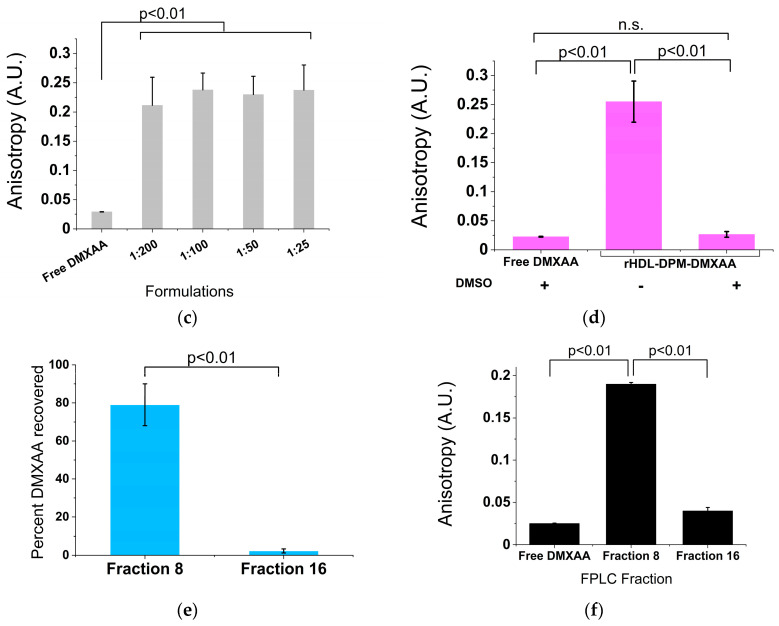
Addition of the DPM enhances DMXAA retention in the particles. (**a**) Entrapment efficiency calculated using Equation (1) n = 6; (**b**) absorption and fluorescence spectra of free DMXAA; (**c**) steady-state anisotropy of DMXAA in the different formulations calculated using Equation (2), n = 6; (**d**) disruption of 1:50 rHDL-DPM-DMXAA NPs with DMSO. DMXAA was dissolved in DMSO to obtain the steady-state anisotropy of free DMXAA in DMSO (first bar in the graph), n.s (non-significant). Then, the steady-state anisotropy of the free DMXAA in DMSO was compared to that of the DMXAA released in DMSO after disruption of the nanoparticles (DMSO: dimethyl sulfoxide), n = 3; (**e**) percent DMXAA recovered (relative to the initial amount of DMXAA injected in the column) in the eluted FPLC fractions of 1:50 rHDL-DPM-DMXAA NPs, n = 3; (**f**) steady-state anisotropy of DMXAA in the eluted FPLC fractions of the 1:50 rHDL-DPM-DMXAA NPs, n = 3. The graphs are presented as mean ± SD.

Adding DPM to rHDL NPs induced at least a 20-fold boost in EE for DMXAA in the rHDL NPs. This increase was characteristic of the 1:200 rHDL-DPM-DMXAA formulation, while no marked additional increase occurred with higher amounts of the DPM (Figure 3a). Thus, rHDL-DPM NPs exhibited about 70% EE with a final DMXAA concentration of about 200 µg/mL. To ensure that DMXAA was associated with the rHDL-DPM NPs, steady-state anisotropy was utilized to monitor DMXAA since it has fluorescent characteristics (excitation 350 nm, emission 415 nm) (Figure 3b). The DMXAA formulated with rHDL-DPM NPs displayed a significantly higher steady-state anisotropy (r~0.23) compared to free DMXAA (r = 0.03 ± 0.0003) (Figure 3c). In addition, disrupting the rHDL-DPM-NPs particles with DMSO restored the anisotropy to the ground level of free DMXAA (Figure 3d). Similar to the DPM, ApoA-I, and the lipids, DMXAA was also detected in Fraction 8 of the FPLC runs (Figure 3e) with a higher anisotropy in the rHDL-DPM NPs eluted in Fraction 8 compared to the DMXAA eluted in Fraction 16 (Figure 3f). These results indicate that the DMXAA is stably associated with the rHDL-DPM NPs.

### 3.4. The rHDL-DPM NPs Prevent Early Payload Release

**Figure 4:**
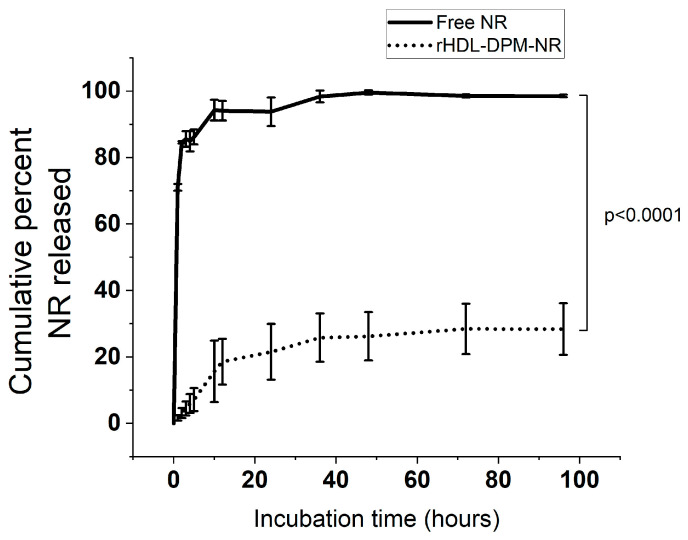
Cumulative percent Nile Red (NR) released over time. The calculations were performed using Equation (3). The data are representative of three independent experiments and are graphed as mean ± SD.

Considering their slightly better physicochemical characteristics than their 1:25 counterpart and having a higher amount of DPM compared to the 1:200 and 1:100, the 1:50 rHDL-DPM NPs were utilized for the subsequent studies. To assess the ability of the particles to retain payload, rHDL-DPM-NR particles were assembled and allowed to release their payload (NR) through a dialysis membrane into the release buffer (PBS). While almost the totality of the free NR was readily released into the buffer during the first few hours of incubation, only 30% of the NR was released by rHDL-DPM NPs at the end of 96 h of incubation (Figure 4). 

### 3.5. The rHDL-DPM-DMXAA NPs Induces Secretion of M1 Cytokines in ID8 CM-Educated RAW 264.7 Macrophages

**Figure 5:**
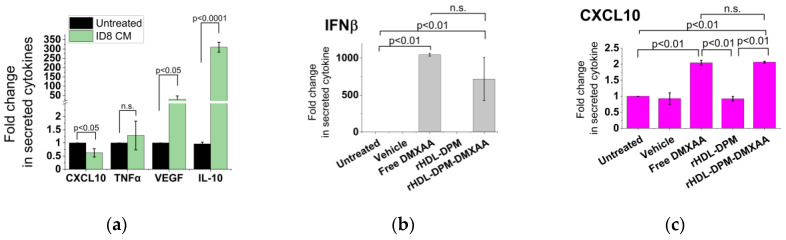

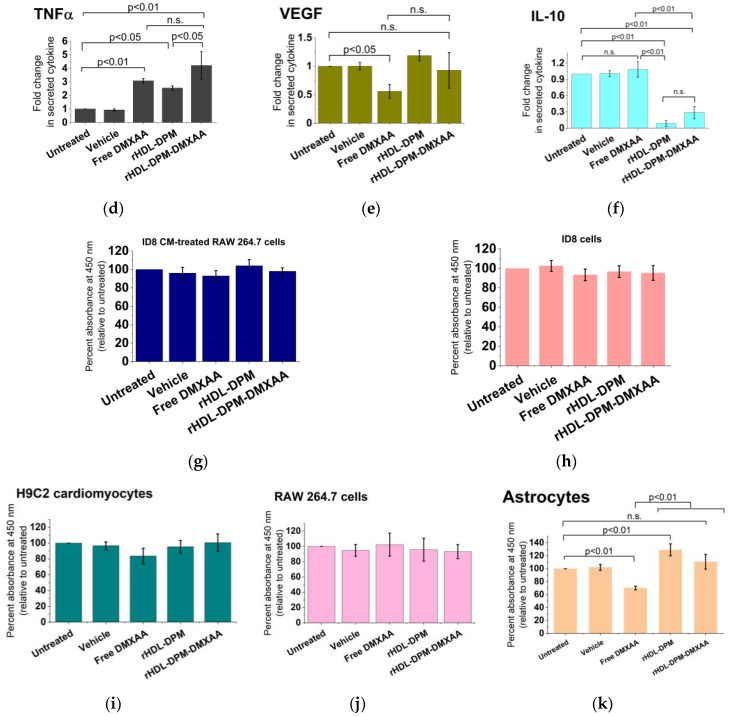
**The rHDL-DPM-DMXAA NPs induce the production of M1 phenotype-related cytokines in ID8 CM-educated RAW 264.7 macrophages.** (**a**) Quantification of secreted CXCL10, TNFα, VEGF, and IL-10 secreted in the supernatant of ID8 CM-educated RAW 264.7 cells via ELISA, n.s. (non-significant); (**b**–**f**) effect of rHDL-DPM-DMXAA NPs and associated controls on the designated cytokine secretion by the ID8 CM-educated RAW 264.7 macrophages; (**g**) assessment of the cytotoxic effect (CCK8 assay) of the rHDL-DPM-DMXAA **1**NPs and associated controls (Equation (4)) on ID8 CM-educated RAW 264.7 macrophages; (**h**) assessment of the cytotoxic effect (CCK8 assay) of the rHDL-DPM-DMXAA NPs and associated controls on the ID8 cells; (**i**) assessment of the cytotoxic effect (CCK8 assay) of the rHDL-DPM-DMXAA NPs and associated controls on the H9C2 cardiomyocytes; (**j**) assessment of the cytotoxic effect (CCK8 assay) of the rHDL-DPM-DMXAA NPs and associated controls on untreated RAW 264.7 cells; (**k**) assessment of the cytotoxic effect (CCK8 assay) of the rHDL-DPM-DMXAA NPs and associated controls on astrocytes. The data are representative of three independent experiments and are graphed as mean ± SD.

In epithelial ovarian cancer (EOC), targeting the prominent M2 immunosuppressive TAMs to promote an M1 phenotype has shown promising results in EOC treatment in clinics [51,52]. Hence, macrophages educated by ovarian cancer constitute a suitable model to assess the M2-to-M1 reversal effect of the rHDL-DPM-DMXAA NPs. Since factors derived from EOC cells have been shown to produce an M2-like pro-tumoral phenotype in TAMs [53,54], RAW 264.7 murine macrophages were exposed to the CM of murine EOC cells (ID8) to create an in vitro model of TAMs. While exposure to ID8 CM slightly decreased levels of secreted M1 phenotype-related cytokine CXCL10, it induced nearly a 50-fold and a 300-fold increase in the secreted levels of the angiogenic factor VEGF and of IL-10 (an M2 phenotype-related cytokine [55]) respectively, in RAW 264.7 macrophages. No significant change was observed for secreted TNFα, also an M1 phenotype-related cytokine (Figure 5a).

Due to the observed M2 phenotype-promoting effect of the ID8 CM on the macrophages and the reported M1-phenotype-promoting effect of DMXAA [56,57,58], we hypothesized that, through the delivery of DMXAA to ID8 CM-educated RAW 264.7 cells, the rHDL-DPM-DMXAA NPs will increase the secretory levels of the M1 cytokines, CXCL10, and TNFα. In addition to inducing the production of the type I interferon IFNβ (Figure 5b) as expected of STING agonists [59,60,61], the treatment of the ID8 CM-educated RAW 264.7 cells with the rHDL-DPM-DMXAA doubled the production of CXCL10 (Figure 5c) and induced about a 4-fold increase in TNFα secretory levels (Figure 5d). Treatment with the rHDL-DPM-DMXAA NPs and the free DMXAA resulted in comparable levels of secreted IFNβ, CXCL10, and TNFα. Interestingly, the rHDL-DPM NPs also elicited TNFα production, albeit to a lower extent than the rHDL-DPM-DMXAA NPs. In contrast to the inhibiting effect of the free DMXAA on VEGF production by the ID8 CM -educated RAW 264.7 macrophages, treatment with the rHDL-DPM-DMXAA NPs did not produce significant changes in VEGF production (Figure 5e). Furthermore, the rHDL-DPM NPs and rHDL-DPM-DMXAA NPs caused at least a 3-fold reduction in IL-10 production, while the free DMXAA did not produce any significant changes in IL-10 production on the ID8-educated RAW 264.7 cells (Figure 5f). The rHDL-DPM-DMXAA NPs did not have a significant cytotoxic effect on either the ID8 CM-educated macrophages (Figure 5j) or the ID8 cells (Figure 5h). Additionally, the survival of non-malignant cells such as the H9C2 cardiomyocytes (Figure 5i), the untreated RAW 264.7 cells (Figure 5j), and astrocytes were not significantly affected by the rHDL-DPM-DMXAA NPs (Figure 5k). Collectively, these data indicate that the rHDL-DPM-DMXAA NPs have the capacity to induce an M1 phenotype in the ID8 CM-educated RAW 264.7 macrophages.

### 3.6. The rHDL-DPM NPs Enhance Payload Uptake in ID8 CM-Educated RAW 264.7 Macrophages

**Figure 6:**
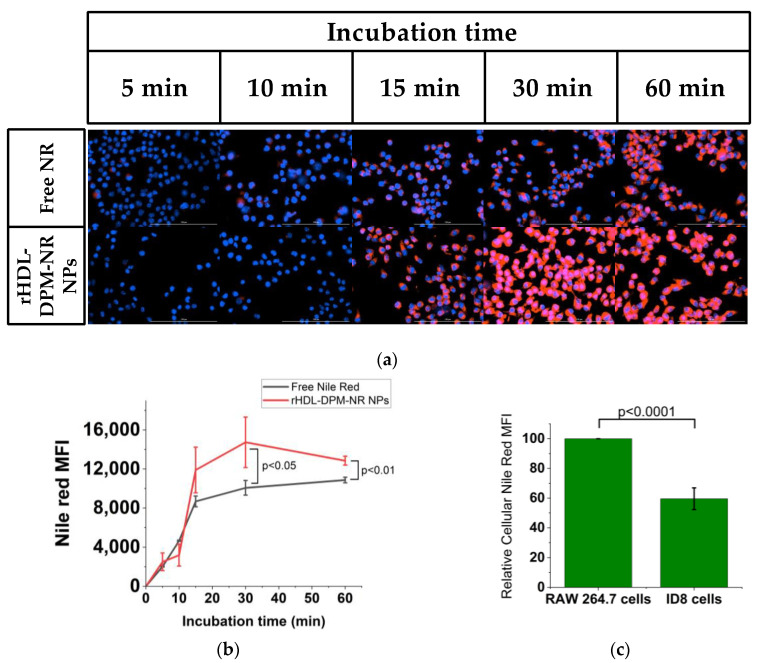

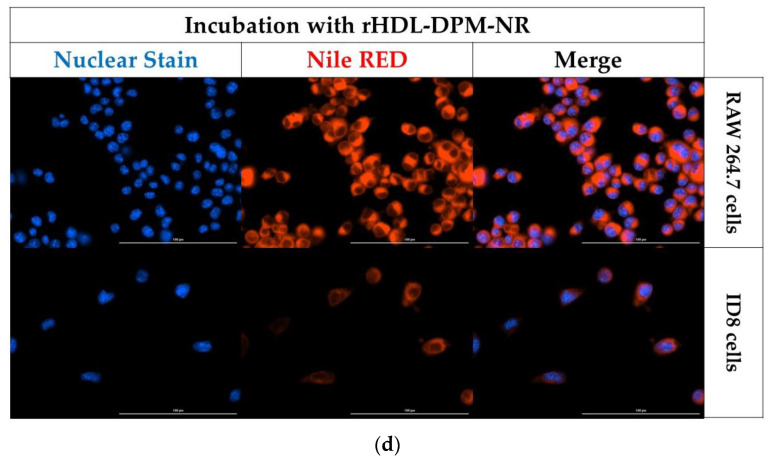
The rHDL-DPM NPs enhance payload uptake in the ID8 CM-educated RAW 264.7 macrophages. (**a**) Kinetics of cellular uptake of NR in the ID8 CM-educated RAW 264.7 cells incubated with NR concentration-matched free NR or the rHDL-DPM-NR NPs. Blue: nuclear stain, Red: Nile Red. Representative images, scale bar: 100 µm; (**b**) quantification of NR MFI from the kinetic study; (**c**) comparison of NR MFI of the ID8 CM-educated RAW 264.7 cells and the ID8 cells incubated with the rHDL-DPM-NR NPs; (**d**) representative images of the NR uptake in ID8 CM-educated RAW 264.7 cells and the ID8 cells incubated with the rHDL-DPM-NR NPs. Blue: nuclear stain, Red: Nile Red. The images and data are representative of three independent experiments, and the data is graphed as mean ± SD.

To evaluate the benefit of utilizing the mannose-coated rHDL NPs in payload uptake by macrophages, the ID8 CM-educated RAW 264.7 macrophages were incubated either with free NR or with rHDL-DPM-NR NPs (NR concentration-matched). The rHDL-DPM NPs mediated a more rapid NR uptake compared to the free NR (Figure 6a,b). In addition, the ID8 CM-educated RAW 264.7 cells exhibited a higher NR uptake than the ID8 cells (Figure 6c,d). These results show that the mannose-coated rHDL NPs promote payload delivery to the ID8 CM-educated macrophages. 

## 4. Discussion

The M2-to-M1 phenotype reprogramming of TAMs accounts for the majority of TAM-centered strategies [62] and can serve as adjuvant therapy to mainstream anticancer therapies [63]. Among the myriads of nanoparticle platforms investigated, the mannose-coated polymers-based carriers or liposomal nanoparticles facilitated TAM targeting in several cancer types [48,64,65,66,67,68,69]. HDL-inspired nanoparticles, including those utilizing ApoA-I mimetic peptides and rHDL NPs, have also been studied and have shown great potential in TAM targeting in the TME [70,71,72]. In the present work, we optimized mannose-coated rHDL NPs loaded with DMXAA and evaluated their effectiveness in mediating an M2-to-M1 reversal in vitro. Since the assembly of rHDL NPs has been extensively optimized in our previous studies [73,74] and by other investigators [31,41,75], we focused on varying the amount of the mannose moiety (i.e., the DPM) to achieve optimal physicochemical characteristics for the mannose-coated rHDL NPs. The changes in hydrodynamic size, zeta potential, FPLC elution patterns, and EE upon introducing the DPM indicate that the DPM has modified the structure and surface of the rHDL NPs and, consequently, their interaction with their environment. The particle size estimation methods utilized in this study clearly show an increase in particle diameter size upon DPM addition to the rHDL NPs. However, the TEM images and the DLS-based number distribution of particle size indicate smaller particle diameter sizes compared to the values produced by DLS-based intensity distribution. The tendency of light scattering methods to skew size measurements to larger particles, although they may represent a minor portion of the nanoparticle population since they move slower than smaller ones, coupled with the anhydrous setting of the TEM may explain the size discrepancies between the different modes of DLS-based measurements and the TEM [76,77]. The increase in hydrodynamic diameter size seen with the rHDL-DPM-DMXAA NPs compared with the rHDL-DMXAA NPs can be attributed to the likely increase of the hydration shell elicited by the numerous hydroxyl groups of the PEG portion of the DPM [78]. The numerous hydroxyl groups of the DPM can also contribute to an anionic coating at the neutral pH and render zeta potential more negative, as observed in the case of the 1:50 rHDL-DPM-DMXAA NPs formulation [79]. The alteration of the FLPC elution profile from the usual pattern described for unmodified HDL nanoparticles [80] upon the addition of the DPM may be resulting from a combined effect of a DPM-mediated increase in the molecular weight of the nanoparticles and perhaps of a change in the interaction of the particles with the column and the mobile phase. 

Considering their ability to transport hydrophobic materials, we hypothesized that the rHDL NPs would efficiently encapsulate DMXAA, a hydrophobic drug (XLogP3-AA: 3.2, PubChem). However, the unmodified rHDL NPs could not accommodate DMXAA (EE%: 3.3 ± 0.65). In contrast to the dismal encapsulation efficiency for DMXAA by the rHDL NPs in this study, Zheng et al. reported a 68.4% entrapment efficiency for DMXAA encapsulated in synthetic HDL nanoparticles [81]. The use of DMXAA covalently linked to cholesterol ester likely facilitates the accommodation of the DMXAA at the core of the particles, as cholesterol esters are highly hydrophobic and constitute the natural payload for HDL nanoparticles [82]. The addition of the DPM to the rHDL NPs greatly enhanced the EE% of the nanoparticles. The ability of DSPE-PEG to improve encapsulation efficiency and stability has also been described in other studies [83,84,85,86]. This phenomenon may be explained by the shielding effect potentiated by the PEG portion of the DSPE-PEG at the shell interface, which may help minimize the loss of payload from the nanoparticles via leakage from the core of the nanoparticles [87]. Steady-state fluorescence anisotropy can be utilized to assess the mobility (free versus bound state) of a fluorophore, and it inversely correlates with the rotational motion of the fluorophore excited by polarized light [88]. The magnitude of the anisotropy increases as the degree of rotational freedom of the fluorophore (here, DMXAA) decreases, and this measurement can be utilized to characterize fluorophore loading in a nanoparticle [89,90]. The 10-fold increase in steady-state anisotropy of DMXAA, which returns to ground levels after disruption of the rHDL-DPM-DMXAA NPs, indicates that the nanoparticles produced a movement-restrictive environment for the DMXAA as it is being transported. In addition to these findings, the co-elution of the rHDL-DPM-DMXAA NPs components (lipids, protein, DPM, and DMXAA) in the same fractions of the FPLC runs demonstrate that the rHDL-DPM-DMXAA NPs were stably assembled. The higher magnitude of the negative zeta potential of the 1:50 DPM:EYPC formulation may indicate suspension stability and a low propensity for aggregation [91,92]. The high DPM retention and the zeta potential results suggest that the 1:50 DPM:EYPC is a solidly optimized formulation of the rHDL-DPM-DMXAA NPs with the synthesis protocol utilized in this study. 

In accordance with the reported immunosuppressive effect of cancer cells on macrophages in the TME [93], incubation of the RAW 264.7 macrophages in ID8 CM resulted in increased production of the immunosuppressive cytokine IL-10 and decreased production of the M1 phenotype-related chemokine CXCL10. The rHDL-DPM-DMXAA NPs were able to convert the ID8 CM-induced immunosuppressive phenotype in RAW 264.7 cells to an immunostimulatory phenotype. This M2-to-M1 phenotype reprogramming is evidenced by the increased production of not only CXCL10 but also of TNFα with the rHDL-DPM-DMXAA NPs treatment. Thus, in contrast to their ability to curb early payload release in the cell-free environment of a buffer solution, the mannose-coated rHDL NPs were effective in delivering DMXAA to the macrophages. Together with the natural intratumoral accumulation of HDL-type nanoparticles [70,94,95], this selective release of the payload to the ID8 CM-educated RAW 264.7 macrophages underscores the potential of the rHDL-DPM NPs in improving therapeutic payload bioavailability at the tumor site. Furthermore, the rHDL-DPM NPs were more efficient at delivering their payload to the ID8 CM-educated macrophages than to the ID8 cells. Hence, the rHDL-DPM NPs are likely to improve the targeting and payload delivery to TAMs. Unlike the free DMXAA, the rHDL-DPM NPs did not cause any significant cytotoxic effect on astrocytes. This finding suggests that the nanoparticles may limit DMXAA delivery to astrocytes, in line with our hypothesis that these nanoparticles will improve selective delivery to macrophages. While the rHDL-DPM-DMXAA NPs uptake mechanisms need to be clarified, the induction of IFNβ secretion exclusively by the free DMXAA and the rHDL-DPM-DMXAA NPs in the macrophages suggests that these mechanisms do not significantly impede the effectiveness of DMXAA in stimulating the STING pathway [60,96].

In addition to serving as a delivery vehicle to macrophages, another facet of the mannose-coated rHDL NPs that warrants further investigation and consideration in their utilization is the modulation of macrophage phenotype. While the free DMXAA reduced VEGF production, the rHDL-DPM-DMXAA NPs had no significant effect on VEGF production by the macrophages, implying that the nanoparticles could be interfering with the VEGF-reducing activity of DMXAA. Interestingly, treatment with the rHDL-DPM NPs doubled TNFα secretory levels and reduced IL-10 production by at least 10-fold in the macrophages. The reduction of IL-10 secretory levels by the rHDL-DPM-DMXAA NPs in macrophages was unexpected since our results with the free DMXAA, and that of other investigators, show that DMXAA does not significantly affect IL-10 production in RAW 264.7 cells [97]. IL-10 is a central mediator of immunosuppression in the TME, which promotes tumor progression and therapy resistance not only in EOC but also in other cancers [98,99,100,101,102]. Hence, this additional feature of rHDL-DPM NPs in the modulation of IL-10 secretory levels points to their suitability as a drug delivery vehicle for TAM targeting in the M2-to-M1 phenotype reversal paradigm.

However, the benefit of selective TAM targeting and phenotype modulation by the rHDL-DPM NPs will need to be confirmed and further investigated in an in vivo and humanized setting and with other therapeutic agents, including human STING agonists. In contrast to the limitations of the two -dimensional in vitro setting of the present study, the presence of physiological barriers, as well as the cellular and non-cellular components of the TME and their potential interactions with the nanoparticles in vivo, may provide a more comprehensive view of the biodistribution and the effectiveness of the rHDL-DPM-DMXAA NPs in TAM targeting and repolarization. In vivo studies involving the rHDL-DPM-DMXAA NPs and other therapy modalities, including immunotherapy and chemotherapy in combination treatments, may provide additional insight into the value of the mannose-coated rHDL NPs as a drug delivery platform to TAMs.

## Data Availability

The data is available upon request from the corresponding authors.

## Supplementary Materials

The following supporting information can be downloaded at: https://www.mdpi.com/article/10.3390/pharmaceutics15061685/s1, Table S1: Characterization of the different formulations of the rHDL-DPM-DMXAA NPs.
