# Supplementary material for: Mannose-Coated Reconstituted Lipoprotein Nanoparticles for the Targeting of Tumor-Associated Macrophages: Optimization, Characterization, and In Vitro Evaluation of Effectiveness
Source: Pharmaceutics. 2023 Jun 8;15(6):1685. doi: 10.3390/pharmaceutics15061685 (PMC10304188; doi:10.3390/pharmaceutics15061685)
Supplement: Supplementary file 1 [file pharmaceutics-15-01685-s001.zip › pharmaceutics-2391086-supplementary.pdf]

pharmaceutics

Supplementary Materials

Mannose-Coated Reconstituted Lipoprotein Nanoparticles for the Targeting of Tumor-Associated Macrophages: Optimization, Characterization, and In Vitro Evaluation of Effectiveness

Akpedje S. Dossou [1], Morgan E. Mantsch [2], Ammar Kapic [1], William L. Burnett [3], Nirupama Sabnis [1], Jeffery L. Coffer [3], Rance E. Berg [1], Rafal Fudala [1,†] and Andras G. Lacko [1,*]

1 Department of Microbiology, Immunology and Genetics, UNT Health Science Center (UNTHSC), Fort Worth, TX 76107, USA; akpedjedossou@my.unthsc.edu (A.S.D.); ammarkapic@my.unthsc.edu (A.K.); nirupama.sabnis@unthsc.edu (N.S.); rance.berg@unthsc.edu (R.E.B.)
2 College of Natural Sciences, University of Texas at Austin, Austin, TX 78705, USA; morgan.mantsch@austin.utexas.edu
3 College of Science and Engineering, Texas Christian University (TCU), Fort Worth, TX 76129, USA; w.l.burnett@tcu.edu (W.L.B.); j.coffer@tcu.edu (J.L.C.)
* Correspondence: andras.lacko@unthsc.edu
† Deceased.

Table S1. Characterization of the different formulations of the rHDL-DPM-DMXAA NPs.

Formulations of DMXAA	Particle Size (nm)	Polydispersity Index	Zeta Potential (mV)	%EE	Particle Concentration (particles/mL)
No DPM	49.5 ± 3.9	0.189 ± 0.030	-20.2 ± 3.9	3.3 ± 0.7	8.5E14 ± 2.9E14
1:200	69.9 ± 8.3	0.170 ± 0.045	-30.1 ± 4.7	62.6 ± 8.1	4.9E14 ± 1.7E14
1:100	107.3 ± 31.6	0.177 ± 0.015	-29.0 ± 4.5	66.8 ± 6.6	6.5E14 ± 2.5E14
1:50	131.7 ± 30.9	0.167 ± 0.010	-36.5 ± 3.6	74.4 ± 7.2	5.6E14 ± 9.7E13
1:25	129.6 ± 30.7	0.182 ± 0.012	-24.8 ± 2.2	70.2 ± 8.2	8.3E14 ± 3.5E14
